# Discriminant Analysis of the Nutritional Components between Organic Eggs and Conventional Eggs: A ^1^H NMR-Based Metabolomics Study

**DOI:** 10.3390/molecules27093008

**Published:** 2022-05-07

**Authors:** Feng Xia, Yanrong Zhao, Meijun Xing, Zhenning Sun, Yizhou Huang, Jianghua Feng, Guiping Shen

**Affiliations:** 1 Department of Electronic Science, Fujian Provincial Key Laboratory of Plasma and Magnetic Resonance, Xiamen University, Xiamen 361005, China; xiafeng@xmu.edu.cn (F.X.); zhaoyanrong.ok@163.com (Y.Z.); 33320201150302@stu.xmu.edu.cn (M.X.); znsun@xmu.edu.cn (Z.S.); jianghua.feng@xmu.edu.cn (J.F.); 2 Nanjing Lvming Ecological Farm, Zhangzhou 363602, China; yzhuang2020@126.com

**Keywords:** metabolomics, nuclear magnetic resonance, magnetic resonance imaging, organic eggs, conventional eggs, nutritional component

## Abstract

The difference of nutrient composition between organic eggs and conventional eggs has always been a concern of people. In this study, ^1^H nuclear magnetic resonance (NMR) technique combined with multivariate statistical analyses was conducted to identify the metabolite different in egg yolk and egg white in order to reveal the nutritional components information between organic and conventional eggs. The results showed that the nutrient content and composition characteristics were different between organic and conventional eggs, among which the content of glucose, putrescine, amino acids and their derivatives were found higher in the organic eggs yolk, while phospholipids were demonstrated higher in conventional eggs yolk. Organic acid, alcohol, amine, choline and amino acids were higher in conventional eggs white, but glucose and lactate in organic egg were higher. Our study demonstrated that there are more nutritive components and higher nutritional value in organic eggs than conventional eggs, especially for the growth and development of infants and young children, and conventional eggs have more advantages in promoting lipid metabolism, preventing fatty liver, and reducing serum cholesterol. Eggs have important nutritional value to human body, and these two kinds of eggs can be selected according to the actual nutrient needs.

## 1. Introduction

Egg, as one of the most conventional foods, as well as one of the best nutrition sources for human being [1,2,3], is rich in protein with high biological value [2,4]. The protein quality of egg protein is second only to breast milk, and is the protein closest to breast milk in nature, almost up to 98% of the egg protein can be absorbed effectively by human body [2,5]. In recent years, nutritional and medical scientists in China and abroad have made some new discoveries of eggs referring to its nutritional values and functions in health care, such as nourishment of brain and improvement of intelligence, protection of liver, prevention and treatment of atherosclerosis, prevention of cancer and delaying of aging [1,5,6,7,8].

Organic eggs, also named as firewood eggs (or native eggs), generally refer to the eggs laid by the hens (native hens or free-range hens) raised by families from the rural areas. Those hens grow in natural environment and take natural food [9]. By contrast, the eggs commonly seen in the markets, are mostly the eggs laid by the hens fed with common commercial feed and caged in high density. It is generally accepted that the quality of eggs produced by organic hens will naturally be better as those hens are raised in natural environment and live on natural foods [10]. The conventional eggs are produced by laying hens growing in the hen farms and fed with commercial diet [9,11], especially some hormones may be added to compound feed so as to accelerate the growth of hens and yield more eggs [12,13]. Therefore, most people are willing to buy these organic eggs even with higher prices [14,15,16,17].

In recent years, the varieties of eggs and their nutritional differences have gradually aroused the attention of researchers and consumers as well. Previous studies show that the content of the nutritional elements such as fat, protein, cholesterol, lecithin, and amino acids in organic eggs were higher than those in conventional eggs [3,18,19]. Furthermore, the content of macro elements such as sodium and potassium [20,21], fatty acids such as palmitic acid and stearic acid [16] contained in organic eggs also covers a higher percentage than that of conventional eggs. Compared with organic eggs, conventional eggs have large egg weigh, more egg white, higher eggshells strength and moisture content, as well as more micronutrients (e.g., Mg, Ca, et al.) [11,13,15,22]. However, a small percentage of studies indicate the opposite or contradictory results. It should be noted that most of the studies focus on comparing the differences of the two kinds of eggs in terms of physical properties, chemical composition of macromolecular substances such as protein, lipid, cholesterol and lecithin [16,23,24]. Hence, it is worth focusing once again that the nutritional value of eggs also depends on the high and low content of small molecular substances such as glucose, amino acids and their composition patterns. The metabolic pathways involved in small molecules are also a favorable breakthrough for studying the nutritional value of eggs, and more accurate guidance can be provided by substances to their effects.

Due to the unique advantages, such as simple sample pretreatment, rapid analysis, synchronous and unbiased detection, rich molecular structure information, noninvasive sample detection, and convenient operation, nuclear magnetic resonance (NMR) spectroscopy has been a powerful analytical technique that is widely used for the identification and quantification of medium and small organic molecules and is particularly useful for the study of complex mixtures from food samples, including food quantitative analysis [25], nutrition and function studies [26], food quality control [27] and production control [28]. In addition, magnetic resonance imaging (MRI) is quite effective in observing the internal parts of the food in three-dimensional space, which can not only help to acquire the abundant data of the inner structure and chemical composition of food, but also test the variation of the internal structure and ingredients as the external environment changes, thus to have a better understanding on the influence of external factors in the physical and chemical properties of food [29]. The combination of NMR technique and pattern recognition method has shown a better prospect in the identification of food source [28] and component differences [25], and has become an important branch of “Food Omics” [30,31].

In the study presented here, global metabolic profiles of organic eggs and conventional eggs were studied and compared by using an untargeted ^1^H NMR-based metabolomics. The aim of this work is to gain more insight into the difference of nutrient composition between organic eggs and conventional eggs in association with nutrient utilization, and to provide a scientific basis for consumers to correctly understand the nutritional value of eggs.

## 2. Results

### 2.1. Metabolic Profiles of Egg Yolks and Whites

**Figure 1:**
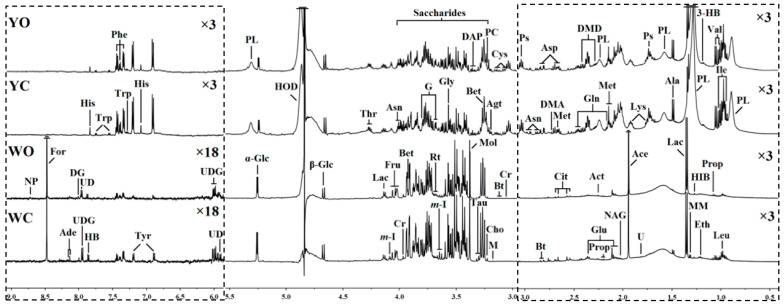
500 MHz ^1^H NMR spectra (δ0.5–3.0, δ3.0–5.5 and δ6.0–9.0) of egg white and egg yolk obtained from conventional and organic eggs. The regions of δ0.5–3.0 and δ6.0–9.0 (in the dashed box) were vertically magnified different times compared with corresponding region of δ3.0–5.5 for the purpose of clarity. YC: egg yolk from conventional eggs; YO: egg yolk from organic eggs; WC: egg white from conventional eggs; WO: egg white from organic eggs.

In order to compare the differences between the two eggs in more detail, the NMR spectra were conducted. Typical 500 MHz ^1^H NMR spectra of egg white and egg yolk samples in organic and conventional eggs were shown in Figure 1. A total of 54 metabolites were are assigned and labeled in NMR egg white and egg yolk spectra by chemical shift, J coupling constant, spectrum peak shape and splitting model, as well as by referring to published literature [32,33,34] and metabolite databases in including HMDB (Human Metabolome Database) and BMRB (Biological Magnetic Resonance Data Bank) [35]. It can be seen from the spectrum that egg white and egg yolk shared similar spectral profiles and they were rich in nutritional substances, such as amino acid, carbohydrates, organic acid, organic amine (alkali), phospholipids, choline and pterin can be detected in the NMR spectra of egg white and yolk.

Keys: 3-HB: 3-hydroxybutyrate; Ace: acetate; Act: acetone; Ade: adenine; Agt: agmatine; Ala: alanine; Asn: asparagine; Asp: aspartate; Bet: betaine; Bt: biotin; Cho: choline; Cit: citrate; Cr: creatine; Cys: cysteine; DAP: 2, 3-diaminopropionate; DG: deoxyguanosine; DMA: dimethylamine; DMD: dimethyladipate; Eth: ethanol; For: formate; Fru: fructose; G: glycerol; Gln: glutamine; Glu: glutamate; Gly: glycine; HB: 4-hydroxybenzoate; HIB: 3-hydroxyisobutyrate; His: histidine; HOD: the residual peaks of water in D_2_O; Ile: isoleucine; Lac: lactate; Leu: leucine; Lys: lysine; M: malonate; m-I: myo-Inositol; Met: methionine; MM: methylmalonate; Mol: methanol; NAG: N-acetylglycoprotein; NP: neopterin; Phe: phenylalanine; PC: phosphocholine; PL: phospholipid; Prop: propionate; Ps: putrescine; Rt: ribitol; Tau: taurine; Thr: threonine; Trp: tryptophan; Tyr: tyrosine; U: unknown; UD: uridine diphosphategalactose; UDG: uridine diphosphate glucose; Val: valine; α-Glc: α-glucose; β-Glc: β-glucose.

As analyze by high-resolution NMR spectra (Figure 1), the egg white and yolk from different groups shared similar spectral profiles but a few differences can be observed by a visual comparison, including the high concentrations of glycine, histidine, tyrosine and in the organic egg yolk, and the high level of choline, leucine, tyrosine, inositol and taurine in the conventional egg white. However, it is difficult to directly compare and analyze the nutritional components of the samples only by visual comparison due to the complex spectrum of eggs. Further investigation of the metabolic profiling of these eggs white and yolk were performed using multivariate analysis techniques.

### 2.2. Comparison of Metabolic Variations between Organic and Conventional Eggs

**Figure 2:**
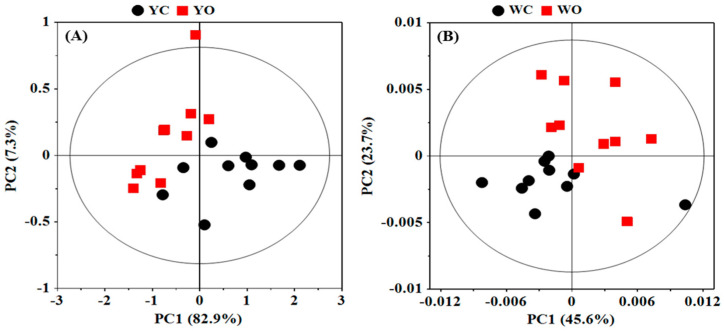
Principal component analysis (PCA) scores plots based on ^1^H NMR spectra of egg yolk (**A**) and egg white (**B**) obtained from conventional and organic eggs. YC: egg yolk from conventional eggs; YO: egg yolk from organic eggs; WC: egg white from conventional eggs; WO: egg white from organic eggs.

A global principal component analysis (PCA) was conducted on the NMR data of egg white and egg yolk in order to display the overall metabolic difference in different egg samples and find the possible outliers (Figure 2). The first two principal components (PCs) explained 90.2% and 69.3% of the total variance in the data of egg white and egg yolk, respectively. PCA scores plots (Figure 2) shown an obvious separation in egg white and egg yolk between organic eggs and conventional eggs, which indicated that there were significant metabolic differences in egg white and egg yolk from different egg.

**Figure 3:**
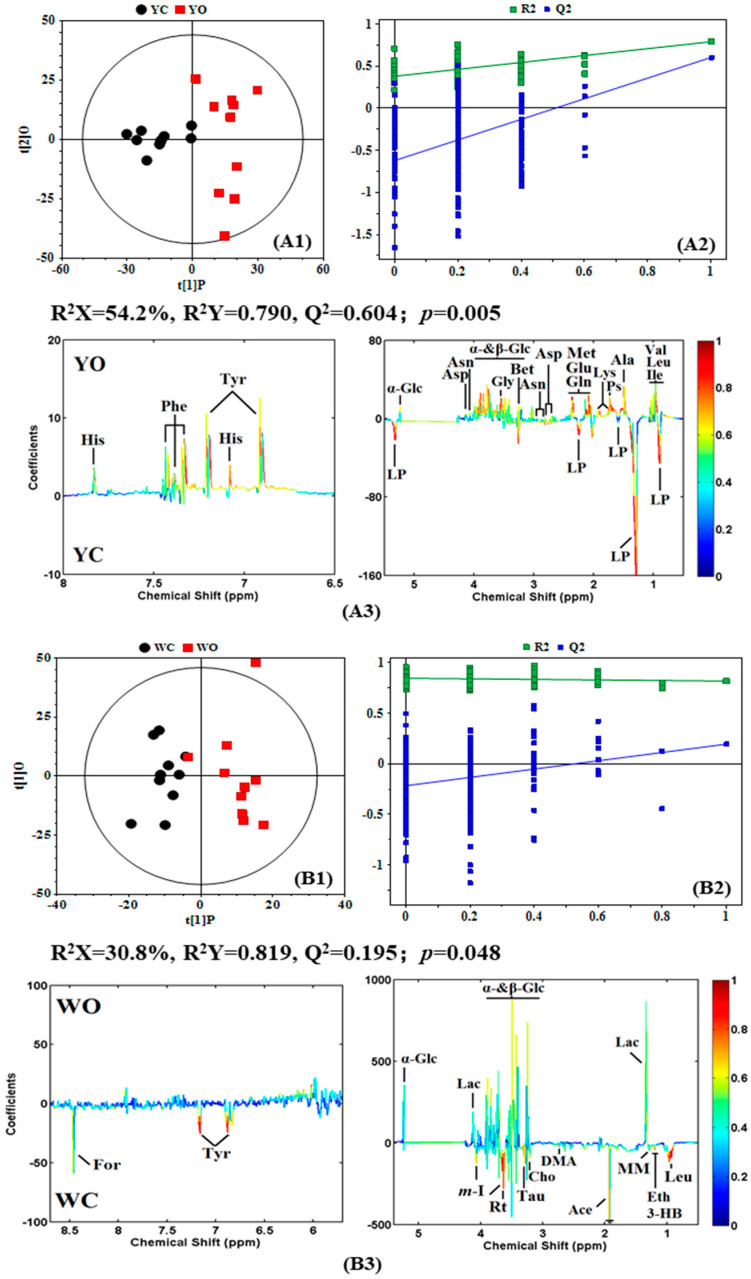
Orthogonal partial least square (OPLS-DA) scores plots (**1**), permutation tests (n = 200) (**2**) and corresponding coefficient loading plots (**3**) derived from ^1^H NMR spectra of egg yolk (**A**) and egg white (**B**) obtained from conventional and organic eggs. YC: egg yolk from conventional eggs; YO: egg yolk from organic eggs; WC: egg white from conventional eggs; WO: egg white from organic eggs. R^2^X and R^2^Y represent the cumulative interpretation rate in the x-axis and y-axis directions, respectively. Q^2^ represents the cumulative prediction rate of the model. The color map shows the significance of nutritional components variations between conventional and organic eggs. Peaks in the positive direction indicate the nutritional components that are more abundant in the organic eggs. Consequently, the nutritional components that are more abundant in the conventional eggs are presented as peaks in the negative direction. Keys of the assignments were shown in Figure 1.

To understand the detailed different metabolic information between organic eggs and conventional eggs, orthogonal partial least square method-discriminant analysis (OPLS-DA) was conducted on the spectral data of egg white and egg yolk from organic eggs and conventional eggs (Figure 3(A1,B1)). The metabolome difference could be derived from NMR data of the different pairwise-comparisons by OPLS-DA. The corresponding coefficient loading plots (Figure 3(A2,B2)) showed the metabolites contributing to the class discrimination, and the correlation coefficients (with color-coded scale) for NMR signals indicated the significance of the metabolites’ contribution, where a hot-colored signal (red) indicates more significant contribution to the class separation than a cold-colored one, and peaks in the positive direction indicate the metabolites that are more abundant in the longer gestation period group, vice versa. In addition, the parameters of Q^2^ and R^2^ are also the indicators of statistical significance of metabolic differences between egg white and egg yolk from organic eggs and conventional eggs. Further analysis found that the R^2^Y and Q^2^ values of the pairwise-comparisons of egg yolk from organic eggs and conventional eggs are 0.790, 0.604, respectively. It is suggested that there is a significant difference in the nutrient composition in egg yolk between organic eggs and conventional eggs, while the difference in the nutritional components of the egg white is not significant (R^2^Y = 0.819, Q^2^ = 0.195). These can be verified by the *p* value obtained from CV-ANOVA, in which *p*-value of the egg yolk and egg white is 0.005 and 0.048, respectively.

Metabolite significant difference in egg yolk and egg white from organic eggs and conventional eggs were assessed using correlation coefficients r and VIP value, i.e., |*r*| > 0.602 and VIP values above top 10% (summarized in Table 1). As shown in Table 1, there are more different metabolites in egg yolk between the organic egg and conventional egg, but less differences in egg whites. The metabolites change in egg yolk and egg white can be summarized as: (1) the level of glucose, amino acids and their derivatives, including alanine, asparagine, aspartate, glutamate, glutamine, glycine, histidine, isoleucine, leucine, lysine, methionine, phenylalanine, tyrosine, valine and betaine, in the organic egg yolk. (2) the level of putrescine is slightly higher in the organic egg yolk, and high level of phospholipid is contained in the egg yolk of conventional egg. (3) some organic acids (e.g., 3-hydroxybutyric acid, acetic acid, formic acid and methylmalonic acid), alcohols (e.g., ethanol, inositol and ribitol) and amine substances (dimethylamine) are higher in the conventional egg white, and choline, tyrosine, leucine and taurine are also higher in the conventional egg white, while glucose and lactate are higher in the organic egg white.

## 3. Discussion

### 3.1. Apparent Properties and Internal Structure of Eggs

As compared with the conventional egg (with an average weight of 55.8 ± 1.7 g), the organic egg is small (with an average weight of 36.2 ± 1.8 g), thin shell, light color. In addition, an organic egg yolk is smaller than a conventional egg yolk with golden yellow and slight red, while conventional egg is light yellow. The egg white in the organic egg is denser than that in the conventional egg. It is showed that both the color of egg yolk and viscosity are important indicators for quality evaluation [12,24]. Darker egg yolk tend to be better, therefore, the quality of organic eggs is higher than conventional eggs in terms of egg physical properties [23]. As investigated by MRI (shown in Figure S1 in the Supplementary Materials), the internal structure and composition of the organic eggs (Figure S1A) and conventional eggs (Figure S1B) can be clearly distinguished, and the combination of the three positions (axial, coronal and sagittal) also provide us with abundant three-dimensional structure information. These results indicate that there is no significant difference in the internal biological structures between the organic and conventional eggs, except that organic eggs have a larger volume percentage of egg yolk than that of conventional eggs [23,36]. Naturally, it is impossible to obtain more detailed nutritional composition information by MRI, so it is necessary to analyze the internal nutritional composition with the help of NMR spectroscopy.

### 3.2. Metabolic and Nutritional Difference of Organic and Conventional Eggs

The nutritional value of protein in food mainly depends on the type and content of essential amino acids [1,2]. In organic egg yolk, the content of amino acid and glucose is higher than that of conventional egg. Especially, histidine, isoleucine, leucine, lysine, phenylalanine and valine, the essential amino acids, are higher in organic egg yolk. Lysine is an essential amino acid for the synthesis of important proteins such as neural regenerative cells, nucleoproteins, and hemoglobin. Lysine not only improves gastric secretion and increases appetite, but also enhances calcium absorption and promotes bone growth and development in children [8,13]. Particularly, it is vital to make supplementation for lysine in infants, so moderate intake of organic egg yolk is quite necessary [6,8,37].

Leucine, isoleucine and valine, three important branched-chain amino acids (BCAA), are significantly higher in organic egg yolks. BCAA are mainly degraded in muscle, kidney, and brain, are involved in the pathways of glycolysis and gluconeogenesis, and can provide important nitrogen sources for other nitrogen-containing substances, such as glutathione, creatinine, creatine, carnitine, pyridine. The previous study have proven that isoleucine increases muscle mass through promoting myogenesis and intramyocellular fat deposition [38] and the BCAA can be served to generate glucose as the first designated substrate of gluconeogenic pathway [39]. In addition, BCAA and aromatic amino acids (phenylalanine, tyrosine) are transported through the blood-brain barrier by a carrier, and they compete with the carrier [7]. When the concentration of branched chain amino acids is high, the entry of aromatic amino acids into brain tissue can be inhibited. Thus, the consumption of organic eggs yolk can provide more energy and help to prevent brain diseases [7,8,13,37].

It should be noted that tryptophan shows a significant high level in organic eggs but not appeared obviously in egg whites. Tryptophan is an essential amino acid that cannot be synthesized by living organisms and needs to be obtained from daily diet. In addition, tryptophan serves as a building block for protein biosynthesis and a precursor for serotonin (a neurotransmitter) [40]. Higher concentrations of tryptophan in the organic egg yolks mean the high nutrition can be received in the daily diet, especially for the infants and pregnant women. Betaine is an efficient methyl donor for the synthesis of methionine and other compounds that play a key role in protein, nucleic acid, and lipid metabolism [41]. This also suggests that the proper consumption of organic eggs yolk can better improve the lipid metabolism, promote the synthesis of protein and improve the development of body.

Meanwhile, organic eggs will be tasted better because of the higher content of flavor amino acids such as glutamic acid and aspartic acid [42], but higher putrescine will lead to a strong fishy smell [43]. Conventional egg yolk contains more phospholipids, which play an important role in activating cells, maintaining metabolism and balanced secretion of hormones, enhancing human immunity and regeneration. It seems that conventional eggs play a greater role in promoting lipid metabolism, preventing fatty liver, lowering serum cholesterol, improving blood circulation and preventing cardiovascular disease [7,13].

Furthermore, in the conventional egg white, the high content of organic acids, such as formic acid, ethanol, alcohols (e.g., ethanol) and amines (e.g., dimethylamine substances) might affect their nutritional value. In addition, there are some beneficial substances such as choline, tyrosine, leucine and taurine in the egg white of conventional egg is higher than that in organic egg, suggesting that people can choose from different aspects for consideration and types of eggs.

All in all, it is appeared that organic eggs have a better advantage over conventional eggs in terms of nutritional content showed by this study under different dietary conditions. In addition, the nutritional differences of eggs may be derived from the differences both in diet and management mode of hens. Therefore, the combined analysis of diet and management of hens may be carried out to acquire more insight into the detailed nutritional differences between conventional eggs and organic eggs.

## 4. Materials and Methods

### 4.1. Sampling Procedure

Ten eggs were randomly sampled from organic and conventional eggs groups, respectively. All the organic eggs (called insects-grass eggs) for the experiment were provided by the LvMing Ecological Farm in Nanjing, Fujian, China, which were produced by hens taking natural food, mainly, some insects, worms, and grass, and foraging for freely in woods, gardens, or orchards. In addition, these hens were fed with diet supplemented scientifically with a certain amount of fly maggots and pennisetum, in order to produce more nutritional eggs [12,44,45]. Certified organic eggs were identified, based on the presence of an organic logo. The conventional eggs were also provided by the same farm and were produced by the same species hens as those produced organic eggs, but these hens were raised in captivity and fed only fresh common feed (Zhengda 324 laying hens feed, Shijiazhuang Zhengda Feed Co., Ltd., Shijiazhuang, Hebei, China) The ingredient compositions and nutrient levels of diet was shown in Table S1 in the supplementary materials. All eggs were cleaned, weighted and stored at 4 °C until analysis.

### 4.2. NMR Experiment

#### 4.2.1. Sample Preparation of Egg White

All chemicals and reagents were purchased from Sinoreagent (Shanghai, China). Egg white was collected using a syringe and placed at room temperature for 5 min. Each egg white sample (30 mL) was vortexed for 60 s in methanol (20 mL) (3:2 *v*/*v*). The mixture was transferred to a 5 mL tube, and centrifuged for 20 min (12,000× *g*, 4 °C). Then, 1 mL upper supernatant was mixed with 3 mL methanol and centrifuged for 20 min (12,000× *g*, 4 °C). After centrifugation, the upper supernatants were transferred to 3 mL tubes, and placed in the vacuum drying oven at 30 °C for 20 min, lyophilized at −80 °C for 24 h at the pressure of 0.01 mbar (FD-1B-80 freeze-drying machine, Jiangsu Tianling Instrument Co., Ltd., Yancheng, Jiangsu, China) to remove methanol and water, then stored at −80 °C for NMR experiments.

The freeze-dried powder of the egg white was collected, mixed with 450 μL double-distilled water and 300 μL of 90 mM sodium phosphate buffer (pH 7.4) containing 0.02% sodium 3-(trimethylsilyl) propionate-2,2,3,3-d_4_ (TSP), an internal chemical shift standard. The extracted egg white buffer mixture was placed at room temperature for 5 min, and then centrifuged at 12,000× *g* and 4 °C for 20 min to remove suspended debris. Then 550 μL of the supernatant was transferred to a 5-mm NMR tube and stored at 4 °C until the ^1^H NMR data acquisition.

#### 4.2.2. Sample Preparation of Egg Yolk

The separated egg yolk was placed into a 50 mL EP tube and lyophilized at −80 °C for 24 h. 450 mg freeze-dried powder of the egg yolk was collected, and mixed with 3 mL double-distilled water. The extracted egg yolk mixture was vortexed for 60 s and placed at room temperature for 10 min, and then centrifuged at 12,000× *g* and 4 °C for 20 min to remove suspended debris. Then 300 μL of the supernatant was transferred to a 1 mL tube with 300 μL of 90 mM sodium phosphate buffer (pH 7.4), and then centrifuged at 12,000 × *g* and 4 °C for 20 min. Then 550 μL of the supernatant was transferred to a 5-mm NMR tube and stored at 4 °C until the ^1^H NMR data acquisition.

#### 4.2.3. Nuclear Magnetic Resonance Spectroscopy

All ^1^H NMR spectra of the egg white and egg yolk samples were acquired at 293 K by using an Agilent NMR System 500 MHz spectrometer (Agilent, Santa Clara, CA, USA) equipped with 5 mm actively shielded x, y, z axis gradients indirect detect probe. Spectra were obtained with a one-dimensional pulse sequence based on a NOESY (nuclear Overhauser effect spectroscopy) pulse sequence (RD-90°-*t*_1_-90°-*t*_m_-90°-Acq) with water suppression (NOESYPR1D). The 90° pulse length was adjusted to approximately 10 μs. The relaxation delay (RD) was set as 5.0 s at a fixed interval *t*_1_ of 4 µs. The water resonance was irradiated during relaxation delay and the mixing time *t*_m_ of 100 ms. The spectral width was set at 6 kHz, and 64 transients were collected into 32 K data points for egg white spectrum, and 32 transients were set for egg yolk. Acquisition time was 2 s and 0.6 s for egg white and egg yolk, respectively.

#### 4.2.4. Preprocessing of NMR Spectra and Multivariate Statistical Analysis

All spectra were pre-processed with the software MestReNova (V8.0, Mestrelab Research S.L.). Prior to Fourier transformation, the free induction decays (FIDs) were zero-filled to 64 K data points and multiplied by an exponential function of 0.3 Hz line-broadening factor. Afterwards, all spectra were phase- and baseline-corrected. Finally, the chemical shifts were referenced to the TSP signal at 0.0 ppm. The spectral regions of the resonance influenced by water (6.50~5.40 ppm), residual water resonance (5.20~4.30 ppm) and TSP signal (0.0 ppm) were removed from the spectra of egg yolk. Each spectrum was then binned into 1100 buckets with intervals of 0.005 ppm across the range of 8.0~0.5 ppm. For the egg white spectra, the spectral regions of the resonance influenced by water (5.65~5.30 ppm), residual water resonance (5.23~4.25 ppm), residual methanol resonance (3.34~3.38 ppm), and TSP signal (0.0 ppm) were removed, then spectra over the range of 8.7~0.5 ppm were binned into 1366 buckets with an interval of 0.005 ppm. To account for overall variations in sample concentration, each spectrum was normalized to its total integrated area.

The NMR spectral data obtained by normalization were imported into the software SIMCA-P software (version14.0, Umetrics AB, Umeå, Sweden) for multivariate statistical analysis. The normalized bucket data were scaled by mean center (Ctr) first and subjected to PCA for the overview of the data distribution and potential outliers. Then, OPLS-DA under a unit variance (UV) scaling pattern were also implemented on NMR data for better understanding of the specific metabolomic difference between the organic and conventional eggs. The pairwise-comparisons were performed and validated with 10-fold cross validation and permutation test (permutation number n = 200) by OPLS-DA methods. Additional validation method, CV-ANOVA was also conducted to validate the models. The quality of the model was assessed by the cross-validation parameter Q^2^, indicating the predictive ability of the model, and R^2^, indicating the total explained variances. In all instances, the Pearson correlation coefficient (*r*) values and the variable importance for projection (VIP) values (the top 10% VIP value) from OPLS-DA models were used to determine metabolites with significant changes. The correlation coefficient loading plot was generated with MATLAB scripts with some in-house modification.

## 5. Conclusions

In this article, NMR-based metabolomics was employed to study the different nutritional composition and composition characteristics in egg white and egg yolk of organic eggs and conventional eggs. The results showed that the nutritional components and composition characteristics between organic eggs and conventional eggs were different, among which the egg yolk of organic eggs contained higher content of glucose, various amino acids and their derivatives and putrescine, while egg yolk of conventional eggs contains more phospholipids. The content of acids, alcohols and amines is higher in the egg white of conventional eggs, as well as choline and certain amino acids. While, the content of glucose and lactate is higher in the egg white of organic eggs. Egg yolk in conventional eggs contains high phospholipids. In general, organic eggs contain higher nutritional value than conventional eggs, which may indicate that organic eggs are relatively better choice for consumers in the daily life.

## Figures and Tables

**Table 1:** Orthogonal partial least square (OPLS-DA) coefficients and VIP value derived from the NMR data of egg yolk and egg white obtained from conventional and organic eggs.

Component and the Corresponding Assignments	YC-YO ^a^		WC-WO	
	*r* ^b^	VIP ^c^	*r*	VIP
3-Hydroxybutyrate: 1.20(d ^d^)	/ ^e^	/	−0.661	1.861
Acetate: 1.92(s)	/	/	−0.614	1.603
Alanine: 1.48(d)	0.955	1.525	/	/
Asparagine: 2.86(dd), 2.97(dd), 3.95(dd)	0.915	1.485	/	/
Aspartate: 2.68(dd), 2.83(dd), 4.00(dd)	0.921	1.481	/	/
Choline: 3.21(s)	/	/	−0.618	1.581
Dimethylamine: 2.73(s)	/	/	−0.760	1.962
Betaine: 3.27(s), 3.91(s)	0.848	1.371	/	/
Ethanol: 1.91(t), 3.65(q)	/	/	−0.749	2.039
Formate: 8.46(s)	/	/	−0.695	1.820
Glutamate: 2.04(m), 2.09(m), 2.36(m), 3.78(t)	0.986	1.572	/	/
Glutamine: 2.14(m), 2.46(m), 3.78(t)	0.952	1.501	/	/
Glycine: 3.57(s)	0.940	1.507	/	/
Histidine: 7.08(s), 7.83(s)	0.930	1.492	/	/
Isoleucine: 0.94(t), 1.01(d)	0.982	1.566	/	/
Lactate: 1.33(d), 4.12(q)	/	/	0.683	1.762
Leucine: 0.96(t)	0.943	1.506	−0.820	2.112
Lysine: 1.73(m), 1.92(m), 3.01(t), 3.75(t)	0.929	1.492	/	/
Methionine: 2.14(s), 2.65(t)	0.950	1.518	/	/
Methylmalonate: 1.28(d)	/	/	−0.610	1.703
*myo*-Inositol: 3.63(t), 4.07(t)	/	/	−0.775	2.003
Phenylalanine: 7.34(d), 7.39(t), 7.44(m)	0.928	1.488	/	/
Phospholipid: 0.89(br), 1.29(br), 1.58(br), 2.24(br), 5.31(br)	−0.909	1.454	/	/
Putrescine: 1.75(t), 3.04(t)	0.874	1.395	/	/
Ribitol: 3.66(m)	/	/	−0.795	2.040
Taurine: 3.28(t), 3.43(t)	/	/	−0.635	1.665
Tyrosine: 6.90(d), 7.20(d)	0.951	1.518	−0.867	1.697
Valine: 0.99(d), 1.04(d)	0.978	1.559	/	/
α-Glucose: 3.42(t), 3.54(dd), 3.71(t), 3.73(m), 3.84(m), 5.24(d)	0.910	1.465	0.680	1.759
β-Glucose: 3.25(dd), 3.41(t), 3.46(dd), 3.49(t), 3.90(dd), 4.65(d)	0.935	1.498	0.751	1.935

^a^ YC: egg yolk from conventional eggs; YO: egg yolk from organic eggs; WC: egg white from conventional eggs; WO: egg white from organic eggs. ^b^ Correlation coefficients, positive and negative signs indicate positive and negative correlation in the concentrations, respectively. The correlation coefficient of |*r*|> 0.602 was used as the cutoff value for the statistical significance based on the discrimination significance at the level of *p* = 0.05 and *df* (degree of freedom) = 9. **^c^** VIP, variable importance. The top 10% of VIP values were selected as the components with statistical significance. ^d^ Multiplicity: s, singlet; d, doublet; t, triplet; q, quartet; dd, doublet of doublets; m, multiples; br, broad resonance. ^e^ ‘‘/’’ means the correlation coefficient |*r*| is less than 0.602 or those VIP not in top 10% of VIP values.

## Data Availability

Data is contained within the article.

## Supplementary Materials

The following supporting information can be downloaded at: https://www.mdpi.com/article/10.3390/molecules27093008/s1, Figure S1: Magnetic resonance (MR) images of the internal biological structure of organic eggs (A) and conventional eggs (B) (1, axial images; 2, coronal images; 3, sagittal images); Table S1: Ingredient compositions and nutrient levels of diet for conventional eggs laying hens.

## Sample Availability

Samples of the NMR and MRI experiments are available from the authors.
