# Supplementary material for: Discriminant Analysis of the Nutritional Components between Organic Eggs and Conventional Eggs: A 1H NMR-Based Metabolomics Study
Source: Molecules. 2022 May 7;27(9):3008. doi: 10.3390/molecules27093008 (PMC9102658; doi:10.3390/molecules27093008)
Supplement: Supplementary file 1 [file molecules-27-03008-s001.zip › molecules-1676691-supplementary.pdf]

Supplementary materials

Discriminant Analysis of the Nutritional Components between Organic Eggs and Conventional Eggs: a ^{1}H NMR-based Metabolomics Study

Feng Xia [1], Yanrong Zhao [1], Meijun Xing [1], Zhenning Sun [1], Yizhou Huang [2], Jianghua Feng [1] and Guiping Shen [1,*]

[1] Department of Electronic Science, Fujian Provincial Key Laboratory of Plasma and Magnetic Resonance, Xiamen University, Xiamen 361005, China; xiafeng@xmu.edu.cn (F.X.); zhaoyanrong.ok@163.com (Y.Z.); 33320201150302@stu.xmu.edu.cn (M.X); znsun@xmu.edu.cn (Z.S.); jianghua.feng@xmu.edu.cn (J.F.)

[2] Nanjing Lvming Ecological Farm, Zhangzhou, 363602, China; yzhuang2020@126.com

* Correspondence: gpshen@xmu.edu.cn; Tel.: +86-592-2180728

1. Magnetic Resonance Image (MRI) Analysis

1.2. MRI Experiment

Before the NMR experiments, three eggs were randomly selected from each group for MRI with an Agilent 7.0 T/160 mm research MRI scanner equipped with a 63/95 mm quad birdcage coil (Agilent, Santa Clara, CA, USA). The axial, coronal and sagittal images were obtained with a pulse sequence of Gradient-Echo-Multi-Slice (GEMS). The repetition time (TR) was set to 8.50 ms, echo time (TE) was 4.40 ms, the field of view (FOV) was 60 × 60 mm^2, and the data matrix size was 256 × 256. One slice with a thickness of 0.74 mm was selected.

2. Results of MRI

In the present study, the organic and conventional eggs were firstly analyzed in terms of internals structure using MRI. The axial, coronal and sagittal MRI images of organic eggs and conventional eggs are shown in Figures 1A and B, respectively. As can be seen from Figure 1, egg is mainly composed of egg white and yolk, which are the main parts of embryo development. The embryonic disk is located in the center of the yolk, and the air cell is located on the side of the larger head of the egg, which makes circulation of air easier.

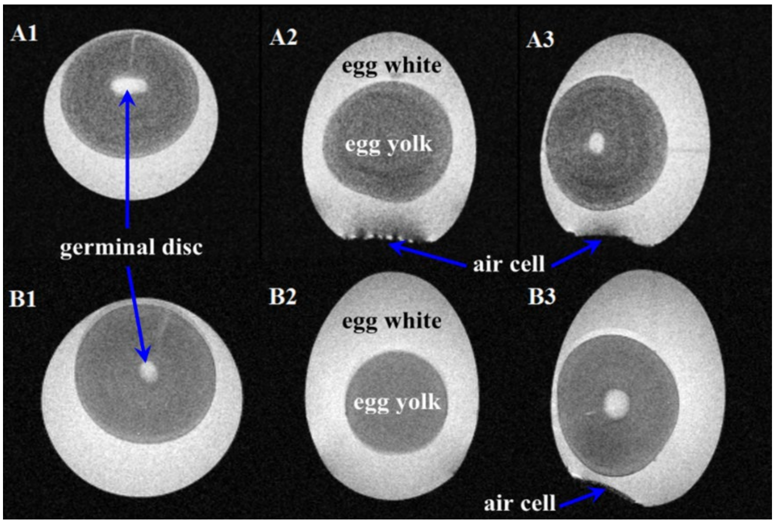

Figure S1. Magnetic resonance (MR) images of the internal biological structure of organic eggs (A) and conventional eggs (B). (1, axial images; 2, coronal images; 3, sagittal images)

Table S1. Ingredient compositions and nutrient levels of diet for conventional eggs laying hens.

Ingredients	Value	Nutrient Level [2]	Value
Corn, %	56.8	Metabolism energy, MJ/kg	10.99
Barley, %	4	Crude protein, %	15.67
Soybean meal, %	19	Crude fat, %	6.00
Soybean oil, %	0.5	Crude fibre, %	6.20
Fish meal, %	2	Ash, %	13.5
Limestone, %	8	Lysine, %	0.80
Di-calcium phosphate	1.35	Methionine, %	0.34
Salt	0.35	Methionine + cystine (%)	0.61
Premix [1], %	8	Calcium, %	3.69
Total	100	Total phosphorus,	0.54

[1] Supplied vitamin and mineral per kilogram of diet: vitamin A 7500 IU, vitamin D3 2500 IU, vitamin E 49.5 mg, vitamin K3 2.5 mg, vitamin B1 1.5 mg, vitamin B2 4 mg, vitamin B6 2 mg, vitamin B12 0.02 mg, Sodium chloride 2500 mg, chloride choline 400 mg, biotin 0.16 mg, pantothenic acid 10 mg, folic acid 1.1 mg, niacin 30 mg, Zn 80 mg, Mn 60 mg, Cu 20 mg, Fe 80 mg, I 0.8 mg, Se 0.3 mg.

[2] Values were calculated from data supplied by the feed database in China.
